# Regulatory issues in Europe

**DOI:** 10.1186/2045-7022-1-S1-S41

**Published:** 2011-08-12

**Authors:** Charlotte B Madsen

**Affiliations:** 1 National Food Institute, Technical University of Denmark, Division of Toxicology and Risk Assessment, Søborg, Denmark

## 

The EU food labelling regulation tells the food producer that all ingredients in a pre-packaged product need to be in the list of ingredients. There are some exceptions from this general rule, but theses exceptions can not be used if the ingredient is a recognised allergenic food on the EU list. It is also important to note that the producer is obliged to use an easily recognisable name. As an example it is not sufficient to write casein on the list of ingredients, because consumers may not know that casein is a milk ingredient. The producer must write e.g. casein (milk). This regulation only covers pre-packaged food and not food sold loose or in restaurants. This type of food is one of the leading causes of severe allergic reactions in people. The European Council adopted in December 2010 an opinion stating that allergen information on non pre-packaged food should be ‘mandatory’. If this proposal is agreed upon by the EU parliament it will be a great advantage for food allergic persons but also a big challenge for small businesses preparing foods sold loose e.g. in bakeries or served in restaurants. Current EU legislation also has not addressed directly the issue of allergen cross contamination. Whilst there is some voluntary guidance which includes qualitative advice for industry on how to assess and manage risk from allergenic foods, there is currently no advice from regulatory bodies or compliance authorities on levels of allergen cross contamination above which advisory labelling (such as ‘May Contain Nuts’) should be used. This current absence of defined thresholds has consequences for risk managers, who have to decide what level of allergenic food in a given product constitutes a health risk and therefore requires action to manage and/or communicate the risk.

